# Reducing the Uncertainty of the Moving Object Location Measurement with the Method of Quasi-Multiple Measurement in GNSS Technology in Symmetrical Arrangement

**DOI:** 10.3390/s23052657

**Published:** 2023-02-28

**Authors:** Jacek Skibicki, Andrzej Wilk, Władysław Koc, Roksana Licow, Jacek Szmagliński, Piotr Chrostowski, Slawomir Judek, Krzysztof Karwowski, Sławomir Grulkowski

**Affiliations:** 1 Faculty of Electrical and Control Engineering, Gdańsk University of Technology, Gabriela Narutowicza Str. 11/12, 80-233 Gdańsk, Poland; 2 Faculty of Civil and Environmental Engineering, Gdańsk University of Technology, Gabriela Narutowicza Str. 11/12, 80-233 Gdańsk, Poland

**Keywords:** quasi-multiple measurements, measurement uncertainty, location measurement making use of GNSS techniques, track geometry measurement, empirical research of rail transport line

## Abstract

The article presents a solution to the problem of limited accuracy of dynamic measurements performed with GNSS receivers. The proposed measurement method is a response to the needs related to the assessment of the measurement uncertainty of the position of the track axis of the rail transport line. However, the problem of reducing the measurement uncertainty is universal for many different situations where high accuracy of positioning of objects is required, especially in motion. The article proposes a new method to determine object’s location using geometric constraints of a number of GNSS receivers arranged in symmetric configuration. The proposed method has been verified by comparing signals recorded by up to five GNSS receivers during stationary and dynamic measurements. The dynamic measurement was made on a tram track within the framework of a cycle of studies upon effective and efficient methods to catalogue and diagnose tracks. A detailed analysis of the results obtained with the quasi-multiple measurement method confirms remarkable reduction in their uncertainty. Their synthesis shows the usability of this method in dynamic conditions. The proposed method is expected to find application in measurements requiring high accuracy, and in case of deterioration of the signal quality from satellites by one or more of GNSS receivers due to the appearance of natural obstacles.

## 1. Introduction

Multiple measurements have been used in metrological practice for a very long time. They consist in repeating the measurement of a given physical quantity and then calculating the result as the arithmetic mean of the data composing the measuring series. The multiple measurements allow to eliminate so-called gross errors and reduce the measurement uncertainty by decreasing the influence of stochastic factors on the measurement process, and finally on the result. In the traditional approach, the multiple measurement is made using the same measuring instrument for a series of successive individual measurements, until a sufficient number of results is obtained to represent a set with stochastic characteristics. It is important for the measurements to be performed in the same physical conditions. However, this procedure limits the use of multiple measurements to measure only the quantities which do not change over time, e.g., mass or dimensions of a physical object [1,2,3,4].

When time-dependent quantities are to be measured, a classical multiple measurement is not applicable. Even if a series of measurements are performed during one session, they are intended rather to demonstrate the course of changes of the measured quantity and, therefore, each measurement in the series is treated individually. In that case, to reduce the measurement uncertainty, a number of measuring instruments should be used to measure simultaneously the same quantity and thus obtain a set of results, a so-called quasi-multiple measurement. In that case the measurement result is the arithmetic mean of the results obtained from individual instruments, according to the formula:

(1) $$x=\frac{1}{n}\cdot \sum_{i=1}^{n}x_{i},$$

where *n* is the number of measuring instruments used.

The standard uncertainty of the measurement of quantity *x* is given by the formula:

(2) $$u\left(x\right)=\sqrt{{\left(\frac{\partial x}{\partial x_{1}}\right)}^{2}\cdot u{\left(x_{1}\right)}^{2}+{\left(\frac{\partial x}{\partial x_{2}}\right)}^{2}\cdot u{\left(x_{2}\right)}^{2}+\cdots +{\left(\frac{\partial x}{\partial x_{n}}\right)}^{2}\cdot u{\left(x_{n}\right)}^{2}},$$

Assuming that all measuring instruments have the same uncertainty, i.e.,:

(3) $$u\left(x_{1}\right)=u\left(x_{2}\right)=\cdots =u\left(x_{n}\right)=u\left(x_{\mathrm{pp}}\right),$$

where *u*(*x*_pp_) is the standard uncertainty of the type of the used measuring instrument, the formula (2) can take the simplified form:

(4) $$u\left(x\right)=u\left(x_{\mathrm{pp}}\right)\cdot \sqrt{{\left(\frac{\partial x}{\partial x_{1}}\right)}^{2}+{\left(\frac{\partial x}{\partial x_{2}}\right)}^{2}+\cdots +{\left(\frac{\partial x}{\partial x_{n}}\right)}^{2}},$$

Since $\frac{\partial x}{\partial x_{i}}=\frac{1}{n}$ then:

(5) $$u\left(x\right)=u\left(x_{\mathrm{pp}}\right)\cdot \sqrt{n\cdot {\left(\frac{1}{n}\right)}^{2}}=\frac{u\left(x_{\mathrm{pp}}\right)}{\sqrt{n}},$$

The disadvantage of multiple measurements is the need to use a number of measuring instruments, which considerably increases the cost of measurement process. Moreover, quasi-multiple measurements can only be applied to measuring quantities for which different location of the measuring instrument (converter) does not affect the measurement result. It might seem that measuring changing location of an object with GNSS antennas does not belong to this group, as locating a number of antennas at the same place in space is impossible [5,6,7]. The method proposed in the article makes use of geometric constraints of a number of GNSS receivers arranged in symmetric 3D configuration, which enables performing a quasi-multiple measurement to determine the location of an object also in dynamic conditions [8,9]. The proposed multi-antenna system was developed for the needs of the project, the aim of which was to determine the position of the track axis in mobile conditions with high precision. For such an application, the use of a single GNSS receiver turned out to be insufficient and did not provide the required measurement accuracy.

The quasi-multiple measurement to determine object location may be of particular use when extreme precision of measurement is required (Precise Point Positioning) or when lower visibility of the constellation of satellites occurs due to, for instance, the presence of natural obstacles [10,11,12,13,14].

Multi-antenna GNSS arrays are known. However, most applications use a cheap miniature devices [15,16,17,18]. This article proposes a multi-antenna system composed of high-end devices to improve positioning accuracy for high-precision applications. This is a kind of novelty in the approach to determining the position of moving objects using the GNSS system. In the classical metrological approach, multiple measurements using high-class GNSS receivers were performed only as so-called long static sessions, intended to determine with extremely high precision coordinates of a given point. In the same way, i.e., using the long session formula, GNSS technology is applied to measure small displacements of building structures, bridge spans, or even the earth’s crust in seismically active regions [19,20,21,22,23]. The measurements executed in the above way, with measuring sessions lasting for many hours, or even days, cannot be used for determining location of a moving object in order to identification of its trajectory with high precision level. Therefore, the proposed method of quasi-multiple measurements is important and necessary, as it provides an opportunity to reduce the uncertainty of measurement in mobile conditions and, what is more, is the only option of measurement in cases when the visibility of satellites is poor.

The proposed method can be used for positioning of all kinds moving objects, both land, sea, and air. It can be used both for the subsequent assessment of travelled trajectory and for feedback systems in controlling of the object position. The latter solution may be useful in precise control of the increasingly used unmanned aerial vehicles or in other applications [24,25,26,27,28].

## 2. Quasi-Multiple Measurements for Satellite-Based Positioning of Physical Object in Dynamic Conditions

**Figure 1:**
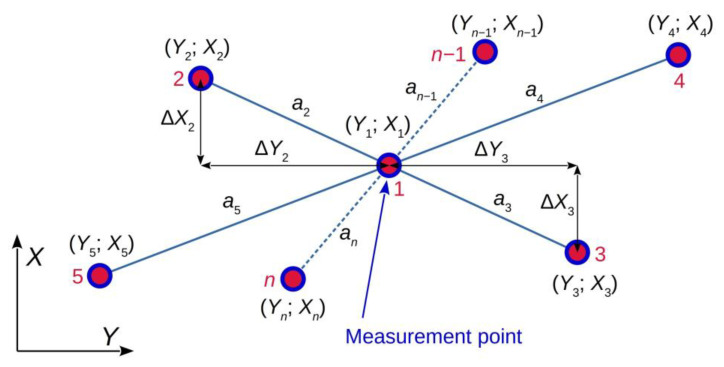
Principle of symmetric 2D configuration of the measuring station for quasi-multiple measurements of physical object location (description in the text).

Due to fact that the reconstruction of rail vehicle trajectory can be conducted separately for plane coordinates of the PL-2000 system and coordinates of the altitude system, further analyses will concern 2D space. The principle of symmetric 2D configuration of the measuring station for quasi-multiple measurements of physical object location in dynamic conditions is illustrated in Figure 1. In the description of this principle, it is assumed that the results of GNSS measurements are obtained in the coordinate system PL-2000, in force in Poland, which is the Gauss–Kruger mapping of the GRS-80 reference system. The GRS-80 system is in practice identical with the WGS-84 system used as the basic reference system in GNSS measurements.

In the example shown in Figure 1, an odd number *n* of GNSS antennas is used. In this case, one antenna, marked 1 in the figure, is installed at the point for which the measurement is to be made, while the remaining antennas are distributed in pairs symmetrically about the measuring point (pairs 2–3; 4–5;…; *n* − 1–*n*). The distribution of pairs of antennas and their distances from the measuring point can be arbitrary, in general with the minimum recommended distance of 1 m. In the configuration shown in Figure 1, antenna 2 measures location coordinates which differ from antenna 1 location by Δ*Y*_2_ and Δ*X*_2_. Similarly, the location of antenna 3 differs by Δ*Y*_3_ and Δ*X*_3_ from antenna 1 coordinates. When the symmetry of antenna locations is preserved, i.e., for *a*_2_ = *a*_3_, the above differences have the same absolute value but opposite sign: Δ*Y*_2_ = −Δ*Y*_3_ and Δ*X*_2_ = −Δ*X*_3_. Similar relations have place for the remaining pairs of symmetrically distributed antennas. Consequently, the coordinates of the measuring point can be calculated as the average mean of location coordinates of all antennas, that is:

(6) $$Y=\frac{1}{n}\cdot \sum_{i=1}^{n}Y_{i},$$

(7) $$X=\frac{1}{n}\cdot \sum_{i=1}^{n}X_{i},$$

The principle of quasi-multiple measurement shown in Figure 1 and described for horizontal coordinates *Y* and *X* is also valid, in the same form, for the vertical coordinate *Z*. This way, the uncertainty level can be reduced for all three coordinates.

For the above procedure to be correct in terms of metrological requirements, the uncertainty of positioning of pairs of antennas with respect to the measuring point (uncertainty of determining the distance *a*) should be an order in magnitude smaller, at least, than the uncertainty of determining coordinates by the GNSS antennas. That means in practice that individual GNSS antennas should be positioned with an uncertainty of the order of one millimetre. When using traditional measuring methods (tape, calliper), this requirement limits the maximal relative distances between the antennas to about few metres, as for larger distances, positioning of the antennas with required precision may turn out difficult from a technical point of view. For road or rail vehicles, longer distances would be difficult to apply due to the dimensions of the vehicle itself. Preserving symmetry and high-precision positioning of GNSS antennas is of high importance, as worsened accuracy in this regard will lead to a positioning error resulting from the fact that the average mean of the obtained results will not correspond with location coordinates of the central point.

When an even number of antennas is used, the distribution principle is almost identical, with the only difference that no antenna is placed at the central measuring point. Regardless of the number of antennas, they must all be of the same type/class.

## 3. Experimental Verification

To verify the above concept of quasi-multiple measurement, location measurements were performed for a two-axle bogie pulled by a tram moving on the track [29,30,31,32,33]. Five GNSS receivers made by Trimble, model R10, and working with frequency 20 Hz have been mounted on the bogie. The Trimble R10 model is based on Trimble HD-GNSS technology which allows measuring point coordinates with high speed and resolution. In this model, the performance and data identification possibility have been increased thanks to the use of an electronic level and tilt compensation, along with positioning at the centimetre level throughout the world using satellite or Trimble CenterPoint RTX internet corrections.

The Trimble xFill technology allows shortening the downtimes resulting from the loss of radio signal. In the R10 model, advanced chipsets GNSS Trimble Custom Survey with 672 channels are used. The list of simultaneously tracked satellite signals includes:

- GPS: L1C/A, L2C, L2E, L5;
- GLONASS: L1C/A, L1P, L2C/A, L2P, L3; SBAS: L1C/A, L5 (for SBAS satellites, which support L5);
- Galileo: E1, E5A, E5B, E5 AltBOC, E61;
- BeiDou: B1, B2, B3;
- QZSS: L1C/A, L1-SAIF, L1C, L2C, L5;
- NavIC (IRNSS): L5;
- Correction services: CenterPoint RTX, OmniSTAR^®^;
- HP, XP, G2, VBS;
- WAAS, EGNOS, GAGAN, MSAS.

To reduce the signal tracking effects caused by high-power out-of-band transmitters, the R10 model has an installed LNA amplifier with 50 dB signal amplification which improves the operation of the equipment in difficult conditions.

The Digital Signal Processor (DSP) technique was applied to detect false GNSS signals and for operation recovery, while the advanced Receiver Autonomous Integrity Monitor (RAIM) algorithm was used to detect and reject questionable satellite measurements and thus to improve the quality of location measurement. The positioning frequencies of Trimble R10 are: 1 Hz, 2 Hz, 5 Hz, 10 Hz, and 20 Hz.

**Figure 2:**
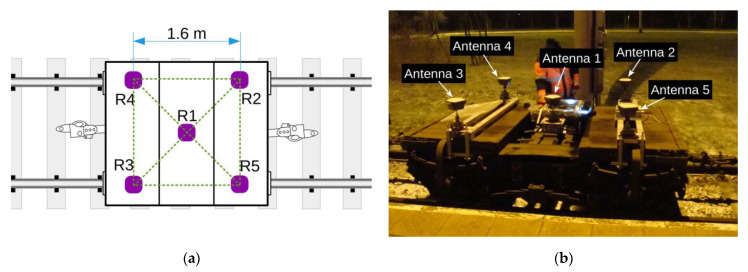
The measuring bogie and the distribution of GNSS antennas: (**a**) distribution scheme; (**b**) view of bogie with antennas.

The raw data obtained from the Trimble model R10 transmitters were complemented with corrections coming from the reference station network ASG Eupos (ASG Eupos is a network of reference stations in Poland). Figure 2 shows the measuring bogie and the distribution of antennas on it.

**Figure 3:**
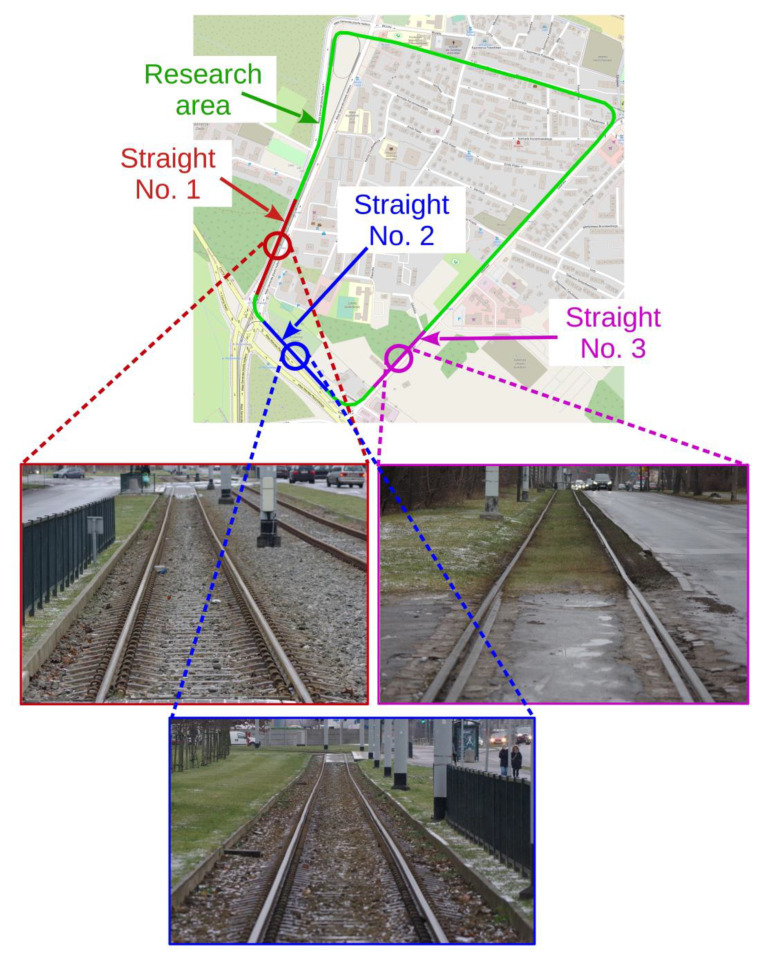
Fragment of Gdańsk tram network with marked zone of measurement and straight-line sections being the object of analysis (Source: own study based on www.openstreetmap.org, accessed on 6 December 2021).

The GNSS antennas are distributed symmetrically at corner points of a square with a side equal to 1.6 m. This arrangement has made it possible to compare the measurement results obtained simultaneously from one to five receivers. The measurements were performed on a selected fragment of Gdańsk tram network which comprised basic geometric structures (straight lines and arcs). To assess the effect of quasi-multiple measurements on measurement uncertainty, a comparison was made between the results obtained in static conditions (during stops) and during the constant-speed motion on three straight line sections situated as in Figure 3.

### 3.1. Results of Static Measurements

The performed series of static measurements has made it possible to assess metrological parameters of the used GNSS receivers related with the uncertainty of location measurement. More precisely, these were not the metrological parameters of the receivers themselves, but rather their current characteristics recorded during the tests which resulted from both their parameters and current availability of the constellation of satellites over the place of measurement, as well as other factors affecting the metrological parameters of the GNSS receivers. These tests provided an opportunity to assess the compliance and degree of identity of accuracy parameters of each individual receiver. For GNSS receivers, the assessment of their parameters based on static measurement is difficult due to occurrence of apparent random displacements, the nature of which resembles the so-called random walking effect [34,35,36], which consists in apparent position change (what is easily observed in stationary measurement session) caused by the action of the algorithms of reference units (here ASG Eupos). This algorithm is intended to improve the accuracy of the measurement results. However this is the source of the abovementioned effect in static measurements. The observed effect of apparent random receiver walk in *YX* plane has the form of uncoordinated trajectory in closest vicinity of its real position. Therefore, a set of measurement results burdened with the effect of random walk is not the set for which the stochastic distribution of measurement results will have properties of a normal distribution [4]. To allow comparing accuracy parameters between individual receivers, the obtained measurement results were subjected to the high pass filtering procedure to eliminate changes corresponding to random walking effect. For this purpose, the random walk signal was first extracted using the third-order low pass filter of rolling average type, with cut-off frequency *f*_g_ = 0.98 Hz. Then, the extracted part was subtracted from the primary signal, thus giving the resultant signal which brings the information about the dispersion of random measurement results. After this operation, it can be assumed that the stochastic dispersion of the obtained set of results has properties of a normal distribution.

**Figure 4:**
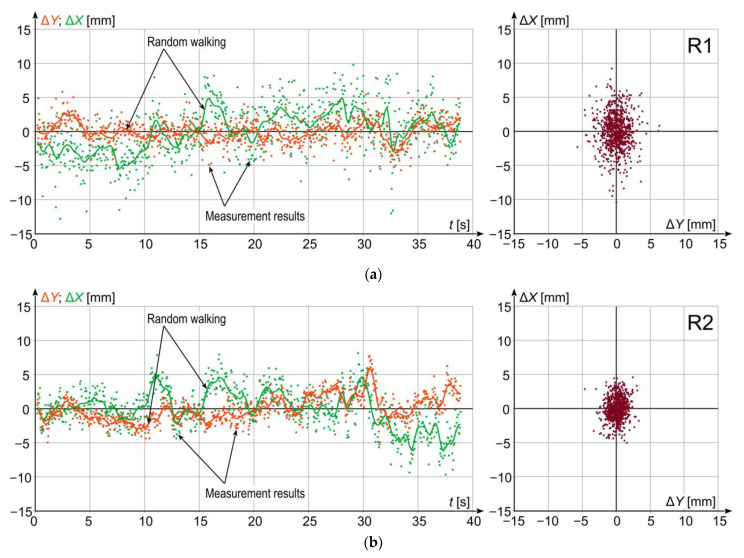

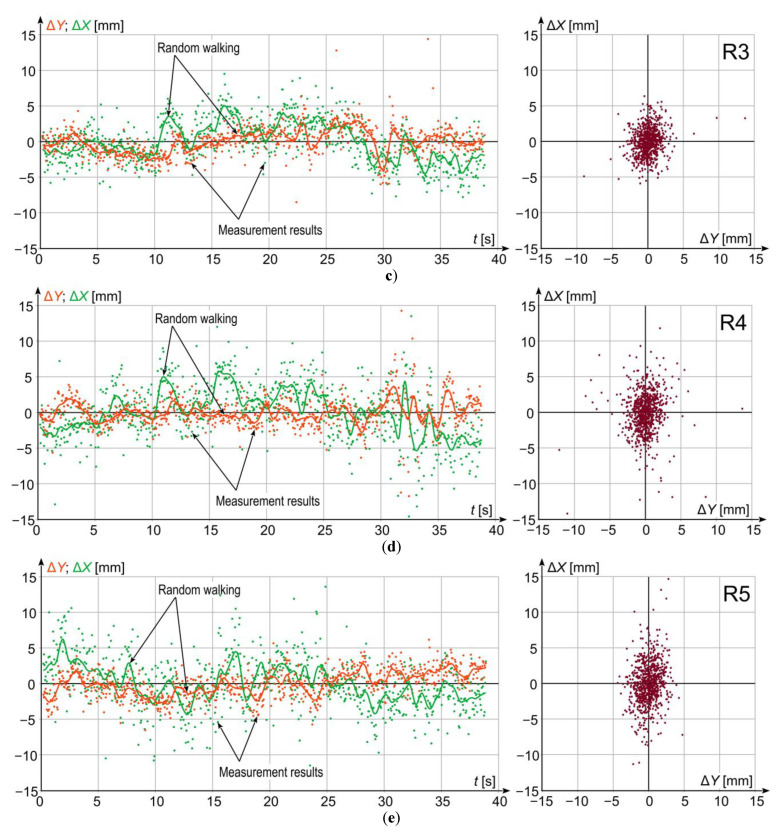
Results of stationary measurements for individual GNSS receivers: (**a**) receiver R1; (**b**) receiver R2; (**c**) receiver R3; (**d**) receiver R4; (**e**) receiver R5.

For a selected tram track fragment, a static measurement was first made, which lasted 39 s. The results of a series of *n* = 780 samples for individual receivers are shown in Figure 4. For each receiver, time-histories are presented with marked random walk trend for *Y* and *X* coordinates, and with the image of stochastic dispersion of the results in *YX* plane after high pass filtering. The standard deviation values of stochastic dispersion for each individual receiver are given in Table 1. The results are presented as differences from the mean of all results obtained.

The results presented in Figure 4 and Table 1 show that during the measurement, the majority of GNSS receivers had similar parameters related with the positioning uncertainty. The resultant standard deviation is about 3 mm. The next step was to analyse standard deviation changes caused by the application of the quasi multi measurement, discussed in Section 2, with simultaneous use of 1, 2, 3, 4, and all 5 GNSS receivers. Since the averaged signals comprise displacements of random walk type as one of components, the resultant signal will also comprise a low-frequency component related with those displacements. Similarly for individual receivers, to remove it, the averaged time-histories were subjected to the high pass filtering procedure, using a filter with the same parameters as for signals from individual receivers. The analysis was performed for the following combinations of receivers:

- Single receiver—receiver 1;
- Two receivers—receivers 2 and 3;
- Three receivers—receivers 1–3;
- Four receivers—receivers 2–5;
- Five receivers—receivers 1–5.

**Figure 5:**
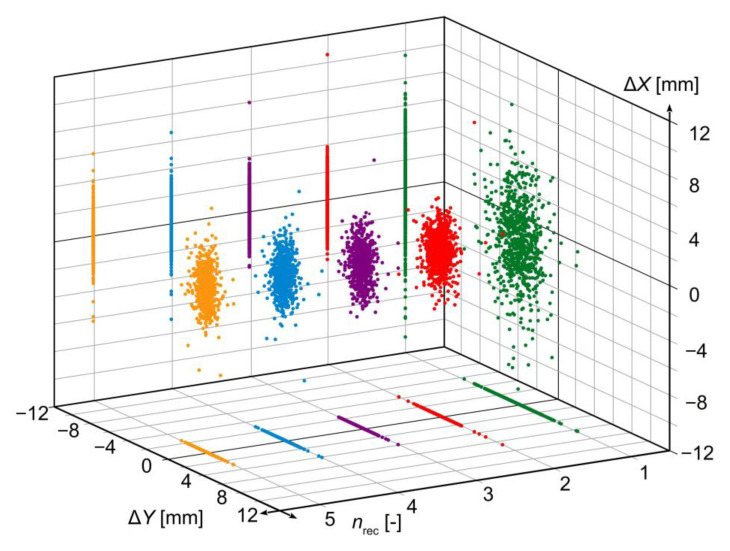
Stochastic dispersion of results for static quasi-multiple measurement vs. number of GNSS receivers used (different colours mean a different number of receivers).

**Figure 6:**
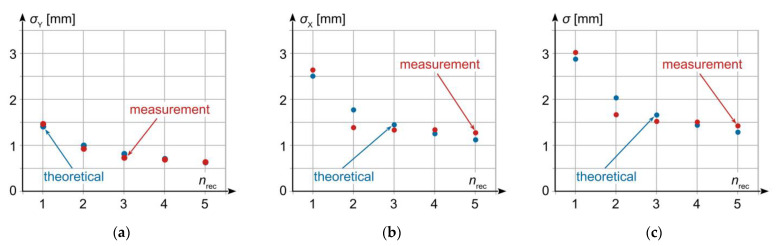
Standard deviation of quasi-multiple measurement results vs. number of GNSS receivers used: (**a**) *Y*-axis; (**b**) *X*-axis; (**c**) resultant.

Stochastic dispersion changes of the results obtained for a given combination of receivers are shown in Figure 5, while the degree of reduction in standard deviation is shown in Figure 6 and Table 2. The obtained standard deviations were compared with theoretical values calculated from formula (5). Since the real standard deviations for individual receivers are not equal, the mean of standard deviations for all receivers was assumed as the baseline level for determining the theoretical value.

The results shown in Figure 5 and Figure 6, and in Table 2 reveal that the reduction level of the standard deviation and, consequently, standard uncertainty of the obtained measurement result is close to that predicted based on the theoretical formula (5). Greater than expected reduction in the uncertainty level for variants making use of two or three receivers is a direct result of better accuracy parameters of receivers 2 and 3 that those of receiver 1, as shown in Table 1. The reason for this, as well as for other differences between the results obtained from measurement and predicted by theory, lies in non-identical metrological parameters of individual GNSS receivers related with different precision of their positioning, which translates into not meeting the condition given in relation (3). Nevertheless, substantial compliance between the theory and measurement is clearly observed, which confirms the validity of the earlier adopted theoretical assumptions concerning the quasi-multiple measurement method.

### 3.2. Results of Measurements Recorded during Bogie Motion

**Figure 7:**
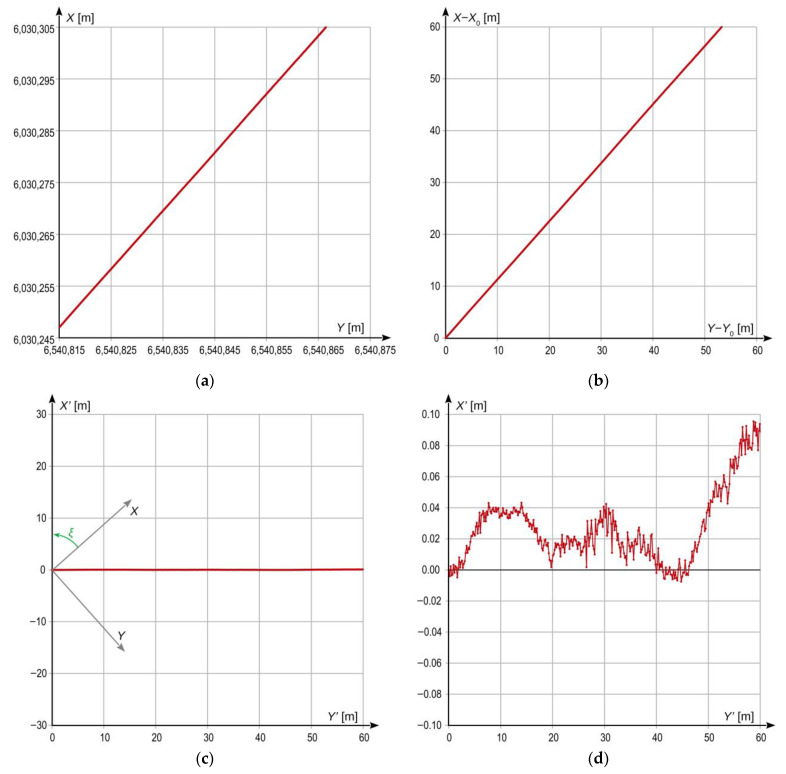
Principle of coordinate system transformation shown based on measurement results obtained on a straight section: (**a**) image in *YX* coordinate system (PL-2000); (**b**) image in *YX* coordinate system with local origin; (**c**) image in *Y*’*X*’ coordinate system rotated by angle *ξ* with respect to *YX* system (isometric view) (**d**) image in *Y*’*X*’ coordinate system in not equal scale of both axis.

**Figure 8:**
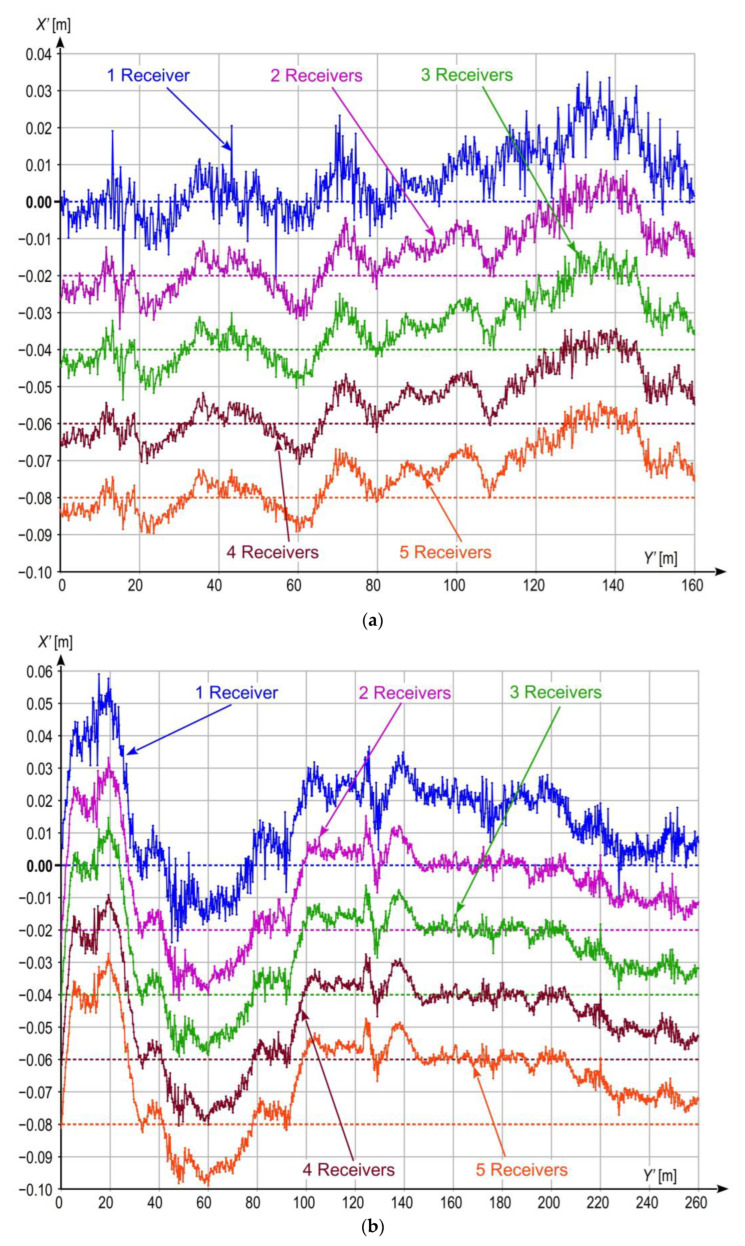

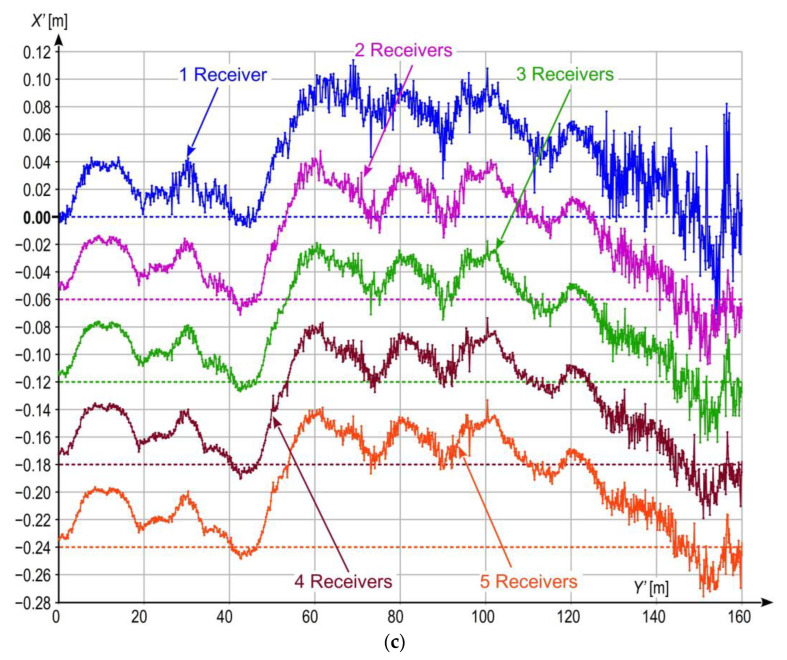
Results of measurements on straight sections, presented in local coordinate systems for 1, 2, 3, 4, and 5 receivers working in the quasi-multiple measurement system: (**a**) Section 1; (**b**) Section 2; (**c**) Section 3.

The dynamic location measurements were performed when the measuring bogie moved along three selected straight tram track sections shown in Figure 3. The lengths of Sections 1 and 2 were equal to 160 m and 260 m, respectively. The track surface of these sections is in very good technical condition, and their horizontal alignment is also very well. They are situated in areas with no large natural obstacles which would potentially disturb satellite signal reception by GNSS receivers. On the other hand, Section 3, with length of 160 m, has been in operation for many years already, and the technical condition of its superstructure is lower than that of the two previous sections. Moreover, the area where Section 3 is situated is occupied by a number of natural obstacles having the form of trees growing near the track. The results of the performed measurements are presented in local coordinate systems *Y*’*X*’ rotated by a given angle (calculated by least square methods from the track axis positions) with respect to the *YX* (PL-2000) system to present local deviations of the tram motion trajectory from a straight line, and to assess the effect of the applied quasi-multiple measurement on the uncertainty (stochastic dispersion) of the obtained measurement results. For this purpose, in each case, the results are plotted in not equal scale of both axes, i.e., in different mapping scales along *Y*’- and *X*-axes. The procedure transforming the coordinate system and its scales is illustrated in Figure 7, and the results of measurements are shown in Figure 8a (Section 1), Figure 8b (Section 2), and Figure 8c (Section 3). Each figure shows the results obtained for one GNSS receiver representing a classic single measurement and for quasi-multiple measurements making use of 2, 3, 4, or 5 GNSS receivers. In order to avoid the overlapping presented signals, the particular plots were offset (along vertical axis) by 0.02 m for Sections 1 and 2, and by 0.06 m for Section 3.

The analysed tram track is not an ideal reference object, as cases of horizontal and vertical unevenness occur along the measured straight sections. The imperfections have been recorded during the bogie motion, and these geometric horizontal track deformations manifest themselves in Figure 8 as waves of a relatively large length (order of several metres) and an amplitude of a few centimetres. The measured parameters correspond to horizontal unevenness of 10 mm in Section 1 and up to 40 mm in Section 3. However, these deviation values are within acceptable limits for trams speed assumed on the measured sections. That means, that the effect of track unevenness of the measuring bogie trajectory can be omitted in the performed analysis.

**Figure 9:**
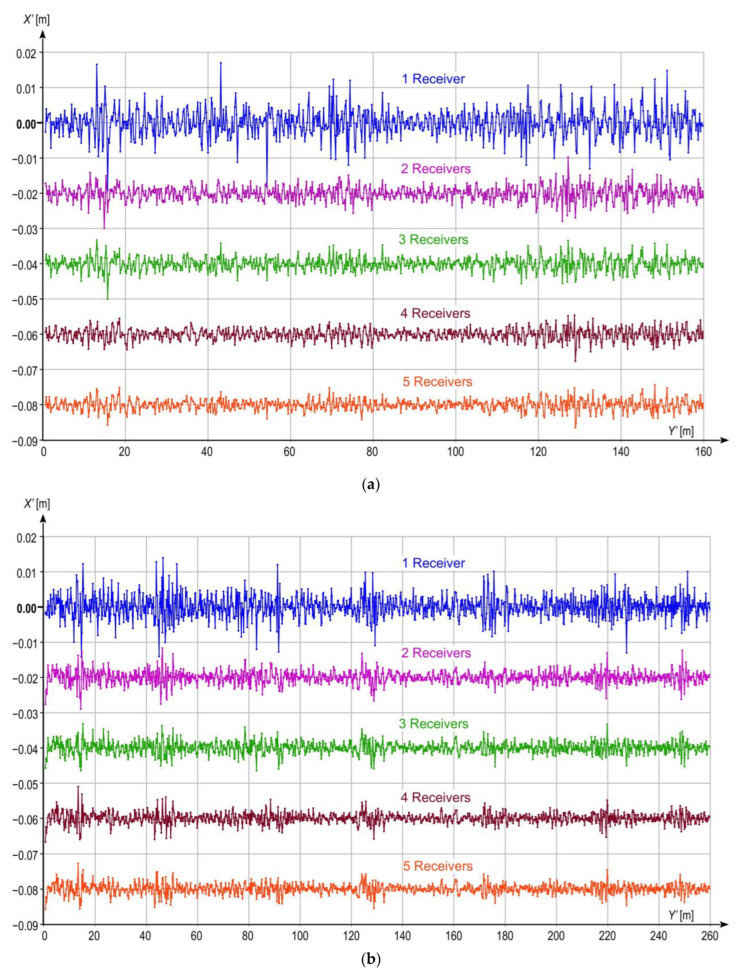

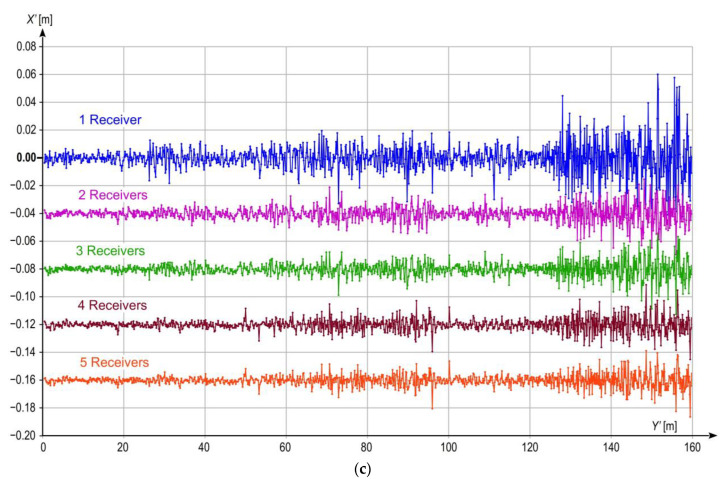
Results of measurements on straight sections after removing slow-changing component, presented in not equal coordinate systems for 1, 2, 3, 4, and 5 receivers working in the quasi-multiple measurement system: (**a**) Section 1; (**b**) Section 2; (**c**) Section 3.

The standard deviation for the fast-changing component of bogie motion along *X*’-axis was adopted as the numerical measure of the measurement inaccuracy reduction level in the analysed system, as it can be assumed that the slow-changing component is caused by local track deformations, while the fast-changing component results from stochastic dispersion of the measurement results. Figure 9 shows the results of measurement for the analysed straight sections, obtained after applying the procedure removing the slow-changing signal component. Similarly as for Figure 8, the results for different solutions were offset by 0.02 m (along vertical axis) for Sections 1 and 2, and by 0.04 m for Section 3. The corresponding standard deviations are given in Table 3. It is worth noting that in this particular case (after removing the slow-changing component), the calculated standard deviation of the X’ coordinates residuals, relative to the mean value will be similar to another method of accuracy assessment, which is the RMSE (root mean square error). In the case of the latter measure, the residuals would be calculated relative to the values determined by the linear model that fits into the series of coordinates (in this particular case, the line would coincide with the horizontal axis Y’). Although the values of the standard deviation and RMSE would be very close to each other, the interpretation of these measures remains different. The shape of the track for a specific model of its axis is not evaluated here, but the uncertainty of measuring the X’ position coordinate of the Y’X’ system. In this sense, measurement uncertainty is calculated using the standard deviation estimator.

The results shown in Figure 9 reveal that in each case, the application of quasi-multiple measurement leads to the reduction of stochastic dispersion of measurement results, which translates into reduction in measurement uncertainty and increase in quality of the obtained results. For Sections 1 and 2, without natural obstacles which would disturb signal reception form a constellation of satellites, the greatest improvement is observed when comparing the results recorded using one receiver with those from two receivers. Further improvement for measurements making use of an increasing number of receivers is smaller, although still visible. Relatively more visible improvement can be observed for Section 3 (Figure 8c), with poor visibility of satellites due to the presence of natural obstacles which translates into larger stochastic dispersion of the results obtained from a single GNSS receiver. In this case, gradual reduction of the stochastic dispersion and, consequently, the uncertainty level is clearly visible for all variants up to four receivers. The quality improvement between variants with 4 and 5 receivers also occurs, but it is very subtle.

The results shown in Table 3 confirm substantial reduction in the uncertainty level when the quasi-multiple measurement method was applied. The obtained percentage reduction in the standard deviation level, compared to single receiver case, is confirmed by theoretical calculations and the results of static measurements (see Table 2). Similarly for static measurements, the observed reduction differences between real results and those calculated from the theoretical formula (5) are caused by differences in characteristics of individual GNSS receivers concerning their positioning accuracy. It is noteworthy, however, that the percentage reduction in uncertainty level for static measurements and for measurements made during bogie motion is similar, compare last columns in Table 2 and Table 3, which indirectly confirms they validity of the methodology adopted for analysing the measurement results.

## 4. Conclusions

The considerations presented in the article confirm the efficiency of the proposed new method of quasi-multiple measurement in reducing the uncertainty level of measurement in dynamic object positioning making use of GNSS technology. This measurement uncertainty reduction is clearly visible regardless of measurement conditions and current accessibility of the constellation of satellites over the area of measurement, although the greatest improvement is obtained for difficult measurement conditions. That shows that the proposed method is especially useful when bad weather conditions and/or the presence of natural obstacles, high buildings or trees for instance, make it impossible in practice to preserve good visibility of the constellation of satellites. It should be remembered that determining of the track axis position should be performed with a precision of at least about 1 cm. The proposed method allows to achieve this condition in localisations where it would be impossible with the use of only one GNSS receiver. Of course, if the GNSS signal is completely lost, e.g., in the tunnel, the measurement will not be possible. Similarly for all quasi-multiple measurement methods, the disadvantage of the proposed method is necessary multiplication of the measuring equipment and the resulting cost increase. On the other hand, the application of the quasi-multiple measurement method may lead to better efficiency of the measurement, along with the reduction in its cost, by eliminating the need for repetition when the quality of the measurement results recorded using a system with one GNSS receiver is unacceptably low. Certainly, the application of the proposed method is not limited to determining the track axis, which is the example shown in the article. Is can be used for arbitrary measurements of objects in motion, or even for stationary measurements, although in this latter case, a possibility to record long measurement series using one instrument makes the use of quasi-multiple measurement less justified. To sum up, the proposed measurement method allows to reduce the uncertainty of dynamic location measurement in *YX* coordinates when using the GNSS technology and provides an opportunity for performing the measurement in difficult conditions, when the presence of natural obstacles worsens the accessibility of the satellite signal. The performed tests have shown that in those cases, the reduction in measurement uncertainty introduced by the quasi-multiple method is especially visible. The disadvantage of the method is the increased costs resulting from the need to use several expensive devices instead of one. However, thanks to this it can be used for measuring location of various types of objects, both mobile and stationary, when an extremely high accuracy of positioning is required.

## Figures and Tables

**Table 1:** Standard deviation of stationary measurement results for individual GNSS receivers.

No. of Receiver	Standard Deviation		
	*σ*_Y_ [mm]	*σ*_X_ [mm]	$\sigma =\sqrt{\sigma_{Y}{}^{2}+\sigma_{X}{}^{2}}$ [mm]
1	1.46	2.64	3.02
2	1.01	1.64	1.93
3	1.44	2.08	2.53
4	1.77	3.18	3.64
5	1.35	2.98	3.27

**Table 2:** Standard deviations of results for static quasi-multiple measurement vs. number of GNSS receivers used.

Number of GNSS Receivers	Standard Deviation						Reduction of Resultant Standard Deviation as Compared to One Receiver [%]	
	*σ*_Y_ [mm]		*σ*_X_ [mm]		$\sigma =\sqrt{\sigma_{Y}{}^{2}+\sigma_{X}{}^{2}}$ [mm]			
	Theoretical	Real	Theoretical	Real	Theoretical	Real	Theoretical	Real
1	1.41	1.46	2.50	2.64	2.87	3.02	0.00	0.00
2	1.00	0.93	1.77	1.38	2.03	1.67	29.29	44.80
3	0.81	0.73	1.45	1.33	1.66	1.52	42.26	49.65
4	0.70	0.69	1.25	1.34	1.44	1.50	50.00	50.14
5	0.63	0.64	1.12	1.27	1.29	1.42	55.28	52.85

**Table 3:** Standard deviations of displacement measurements along *X*’-axis after removing slow-changing component for straight sections for which the quasi-multiple measurement was applied, as function of the number of GNSS receivers used.

Number of GNSS Receivers	Standard Deviation σ along *X*’-axis [mm]			Standard Deviation Reduction as Compared to one Receiver [%]			
	Section 1	Section 2	Section 3	Section 1	Section 2	Section 3	Average
1	3.97	3.22	10.46	0.00	0.00	0.00	0.00
2	2.14	1.85	5.67	46.21	43.47	45.81	45.16
3	1.95	1.70	5.23	51.04	47.21	50.03	49.43
4	1.65	1.53	4.67	58.54	52.50	55.41	55.48
5	1.56	1.45	4.20	60.70	54.87	59.88	58.48

## Data Availability

The obtained measurement data is confidential and therefore is not on a publicly accessible server.
